# Risk Assessment of Face Skin Exposure to UV Irradiance from Different Rotation Angle Ranges

**DOI:** 10.3390/ijerph14060606

**Published:** 2017-06-06

**Authors:** Fang Wang, Qian Gao, Yan Deng, Rentong Chen, Yang Liu

**Affiliations:** 1 School of Public Health, China Medical University, Shenyang 110122, Liaoning, China; wangf@cmu.edu.cn (F.W.); qgao@cmu.edu.cn (Q.G.); chenrentong91@126.com (R.C.); 2 Chinese Journal of Health Statistics Magazine, Shenyang 110122, Liaoning, China; dengyan@cmu.edu.cn

**Keywords:** risk assessment, UV irradiance, biologically effective, skin damage

## Abstract

Ultraviolet (UV) is one of the environmental pathogenic factors causing skin damage. Aiming to assess the risk of face skin exposure to UV irradiance from different rotation angles, a rotating model was used to monitor the exposure of the skin on the face to UV irradiance, with skin damage action spectra used to determine the biologically effective UV irradiance (UVBE_skin_) and UVBE_skin_ radiant exposure (HBE_skin_) causing skin damage. The results indicate that the UVBE_skin_ is directly influenced by variations in rotation angles. A significant decrease of approximately 52.70% and 52.10% in UVBE_skin_ was found when the cheek and nose measurement sites was rotated from 0° to 90°, while a decrease of approximately 62.70% was shown when the forehead measurement sites was rotated from an angle of 0° to 108°. When HBE_skin_ was compared to the exposure limits (ELs; 30 J·m^−2^), the maximum relative risk ratios (RR) for cheek, nose, and forehead were found to be approximately 2.01, 2.40, and 2.90, respectively, which were all measured at a rotation angle of 0°. The maximal increase in the percentage of the average HBE_skin_ for rotation angles of 60°, 120°, 180°, and 360° facing the sun to ELs were found to be approximately 62.10%, 52.72%, 43.43%, and 26.27% for the cheek; approximately 130.61%, 109.68%, 86.43%, and 50.06% for the nose; and approximately 178.61%, 159.19%, 134.38%, and 83.41% for the forehead, respectively.

## 1. Introduction

Ultraviolet (UV) irradiance is one of the important physical factors in the environment and one of the environmental pathogenic factors causing skin damage in humans, which is supported by sufficient experimental and epidemiological evidence [1,2,3,4,5,6,7,8,9]. With the depletion of ozone, increasing life expectancy and the change in modern lifestyle may further increase this UV-related skin damage [10,11,12,13,14,15]. Skin cancer is the most serious skin damage due to UV exposure; 90% of new cases may be attributable to UV exposure [16]. Currently, the number of new cases of skin cancer is 2–3 million a year, with a growing trend all over the world [17]. Skin cancer has become the fourth most common cancer [18,19]. UV-induced skin damage has become an important and alarming issue around the world. Therefore, assessing the risk of skin exposure to solar UV radiation (UVR) to improve risk cognition of UV irradiance is very important for preventing skin damage.

The reliability and accuracy of any risk assessment or hazard evaluation of UV irradiance depend strongly upon the precise quantification of the skin exposure to solar UV radiation, so appropriate metrologies have been developed to measure skin exposure to UV irradiance. The skin exposure to UV radiation is influenced by facial anatomy. A few studies have attempted to measure personal UV exposures during normal daily activities of specific anatomical sites, using human subjects wearing instruments (such as UVR-sensitive polysulfone film, UV-Biometer model 501, electronic personal dosimeter (X-2000), polysulphone dosimeters and et al.) located at specific anatomical sites (for example, shoulders, heads, chest, back, neck, face, and arm) [20,21,22,23,24,25,26,27,28,29,30,31]. Studies have also been conducted using models to simulate specific human anatomical sites for UV irradiance exposure [32,33,34]. On the other hand, human outdoor activities typically occur randomly and are orientated at different directions toward the sun, while workers are instructed to work at more predictable rotation angle ranges relative to the position of the sun in the sky. The angle that an individual faces with regards to the sun is important as it affects skin exposure, although this is also influenced by their activities. Therefore, the effect and the quantification of the UV exposure based on the rotation angle ranges cannot be investigated.

Another factor which influences the reliability and accuracy of risk assessment for skin exposure to solar UV radiation is the precision and accuracy of the relevant action spectra employed and of exposure limits (ELs). The International Commission on Non-Ionizing Radiation Protection (ICNIRP) proposed skin damage action spectra in 2004 and recognized ELs [35] that determine that the biologically effective UV irradiance (UVBE) exposure in the spectral region of 180 to 400 nm on an unprotected skin should not exceed 30 J m^−2^ [35]. These ELs have been used as guides in the control of exposure to UV sources and were developed by considering lightly pigmented populations (i.e., white Caucasians) with greatest sensitivity and genetic predisposition for skin cancer. Exposure during sun bathing and tanning under artificial sources may well exceed these limits, but guidelines on limits of exposure to UV radiation exposed individuals have advised that some health risks are incurred from such an activity.

To the best of our knowledge, a model has not been used to assess the potential health risk of skin exposure to UV radiation at different rotation angles. Taking into account that the face has the highest risk, the face being almost two to four times more sensitive than limbs [36,37,38], the current study measured the face skin exposure to UV spectrum irradiance at different rotation angles using a spectroradiometer and a mannequin. The measured UV spectrum data was weighted by the skin damage action spectrum [35], which has been previously proposed by ICNIRP to calculate the biologically effective UV irradiance for skin damage (UVBE_skin_). The UVBE_skin_ was weighted by the exposure time (T) to calculate the biologically effective UV radiant exposure for skin damage (HBE_skin_). Finally, we calculated the relative risk ratio of HBE_skin_ to ELs to assess the potential risk effects to skin caused by UV radiation. This study could be used to enhance public awareness regarding the risk of skin UV exposure at different rotation angles as well as promoting the use of sun protection measures to reduce potential risk of skin damage caused by UV radiation.

## 2. Materials and Methods

### 2.1. Experimental Setups

**Figure 1:**
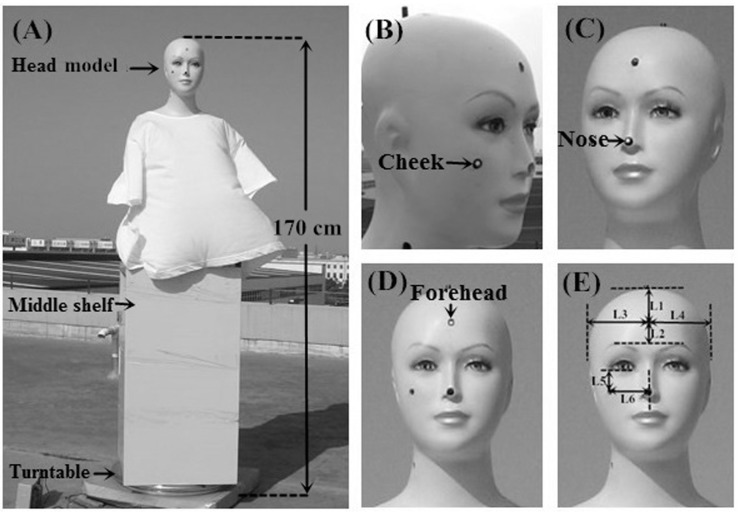
The anatomic measurement sites on model’s head: (**A**) model system; (**B**) cheek; (**C**) nose; (**D**) forehead; (**E**) measurement sites positioning.

From top to bottom, the experimental setup consisted of a head model, a shelf, and a turntable base that rotated at a constant speed. The total height of this model system was approximately 170 cm (Figure 1A). A computer and a computer-controlled fiber-optic (FO) spectrometer with two detectors were placed on the shelf to measure the UV spectral irradiance (in μW cm^−2^ nm^−1^). One detector was placed at the vertex of the model’s head to measurement the horizontal ambient UVR radiation, while the other detectors were placed on the cheek (Figure 1B), nose (Figure 1C), and forehead (Figure 1D). These points were chosen as they hold the plane tangent to the anatomic measurement sites at the most anterior points. The distance between the actual measurement sites at the cheek and the lower eyelid of the model is approximately 2 cm (Figure 1E(L5)), while the distance between the cheek and the nasal septum is approximately 4.5 cm (Figure 1E(L6)). The tip of the nose was the actual anatomic measurement location on the nose. The distance between the actual measurements at the forehead and the vertex of the head models is approximately 5 cm (Figure 1E(L1)), the distance between the forehead and the connection of two eyebrow ridges approximately 2.5 cm (Figure 1E(L2)), and the distance between forehead and the right and left of the head models is 7.5 cm (Figure 1E(L3) and Figure 1E(L4)). The detectors simultaneously recorded the UV radiation levels at the corresponding horizontal ambient and measurement sites.

The spectrometer and equipment calibration used in this study have been described in [33].

The UV radiation measurements were performed in the town of Dou Men near Shao Xing city (30.09° N, 120.60° E, altitude of 553 m), Zhejiang, China. The experimental model system was placed on the roof of a five-story house with asphalt covering the concrete. The measurements were performed between sunrise and sunset on days with a clear or slightly cloudy sky. The cheek and horizontal ambient UV irradiance measurements were performed on 27 May 2010 (summer) from 6:00 to 16:00 China Standard Time (CST), while the nose, forehead, and corresponding horizontal ambient UV irradiance measurements were performed on 30 May 2010 (summer) from 7:00 to 18:00 CST. For both measurement days, solar noon both occurred at approximately 12:00 CST, the midday maximum solar elevation angles (SEAs) were both approximately 82°, and the mean air pollution index (API) was approximately 62. The total column ozone amounts for 27–30 May 2010 were 276 Dobson Units (DU) and 326 DU, respectively.

**Figure 2:**
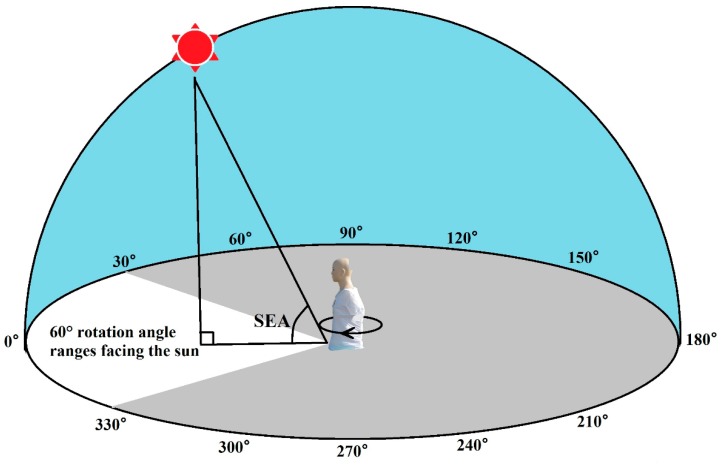
UV measurements for different rotation angles. Notes: Rotation angle of 0° is the initial position of the rotating model facing the sun. The rotation angles are increased clockwise. In this figure, we show 12 rotation angles at 30° intervals. Based on the rotation angles, the rotation angle ranges were determined. For example, the 60° rotation angle range facing the sun was comprised of the rotation angles from 330° to 30° rotated clockwise, shown in white in this figure. The solar elevation angle (SEA) is the angle complementary to the solar zenith angle. The solar zenith angle is the angle between the line of the sun and the model with the line vertical to the ground.

The model was rotated clockwise at a constant speed for 360° over 1 min (equivalent to 6°/s). The duration of each spectrometer measurement was 1 min, and both detectors collected data once per second. Subsequently, 60 sets of facial UV measurements and horizontal ambient UV irradiance per model revolution were collected. The UV irradiance at 1 s intervals was measured over the range of 300–400 nm at 1 nm intervals. The time between measurements was 15 min. For each measurement cycle, as shown in Figure 2, the model began facing towards the sun, which was determined as a rotation angle of 0°. The opposite direction to the sun was determined as a rotation angle of 180°. In this study, we also determined the rotation angle ranges. For example, the 60° rotation angle range facing the sun, in Figure 2, is shown in white, which is comprised of the rotation angles from 330° to 30° clockwise. Similarly, the 120° and 180° rotation angle ranges facing the sun were comprised of the rotation angles from 300° to 60° and 270° to 90° clockwise, respectively. Meanwhile, the 180° rotation angle range backing to the sun consisted of a range from 90° to 270° clockwise.

### 2.2. Data Processing

**Figure 3:**
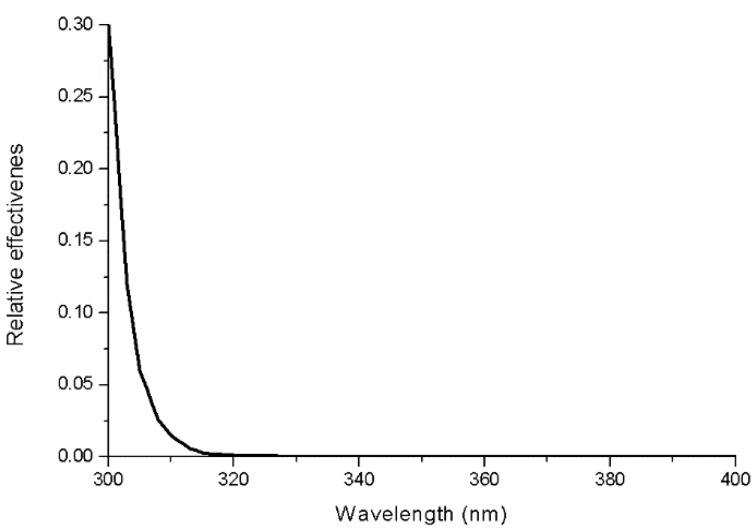
Skin damage action spectra [35].

The UVR and UVBE_skin_ irradiances were calculated according to Equations (1) and (2), respectively, as follows:

(1) $$\mathrm{UVR}=\int_{300}^{400}S(\lambda )d(\lambda )$$

(2) $${\mathrm{UVR}}_{\mathrm{skin}}=\int_{300}^{400}S(\lambda )A(\lambda )d(\lambda )$$

where UVR is the UV irradiance in the 300–400 nm band in μW cm^−2^; UVBE_skin_ is the skin damage biologically effective UV irradiance (300–400 nm band) in μW cm^−2^; *S(λ)* is the measured UV spectral irradiance in μW cm^−2^ nm^−1^; *A(λ)* represents the skin damage action spectrum, and ICNIRP gives the reference for this [35]; and *d(λ)* is the wavelength increment of the UV spectral data (in this case, 1 nm). For this study, the action spectra for skin damage from 300 to 400 nm have been employed, and the action spectra were linearly interpolated between data points to 1 nm (Figure 3). 

The UV radiant exposure (H in J m^−2^) for specific time intervals was calculated according to Equation (3):

(3) $$\text{H}=\int_{T1}^{T2}Ed(T)$$

where *E* represents UVR and UVBE_skin_ irradiance, *T1* is the beginning time, *T2* is the time, and *d*(T) is the time interval (in this case, 15 min). UVR and UVBE_skin_ irradiance weighted by *d*(T) was used to calculate H_UVR_ and HBE_skin_, respectively. The 1 h cumulative H_UVR_ and HBE_skin_ in 60 rotation angles were calculated in this study.

The relative risk ratio (RR) was calculated according to the following equation:

(4) $$\text{RR}=\frac{HBE_{skin}}{30}$$

where HBE_skin_ represents effective radiant exposure in J m^−2^, and 30 represents the exposure limit (ELs) in J m^−2^. According to the ELs published by ICNIRP in 2004 [35], 8 h cumulative HBE_skin_ should not exceed 30 J m^−2^. The relative risk ratio corresponding to 1 h cumulative HBE_skin_ was calculated in this case.

RR ≥ 1: increased risk of skin damage among those that have been exposed to UV irradiance.

RR < 1: association between skin damage and exposure to UV irradiance unlikely to exist.

## 3. Results

### 3.1. Variations of the UVR at Different Rotation Angles

**Figure 4:**
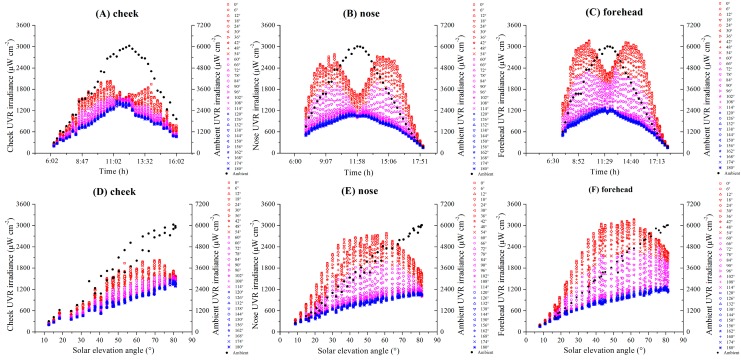
Diurnal variations of UV radiation (UVR) in different rotation angles and UVR changes with SEA. Notes: Since the distributions were approximately axisymmetric along the 0–180° direction, we reported the results from 0° to 180° rotation angles in intervals of 6°. (**A**), (**B**), and (**C**) show the diurnal variations of the UVR irradiance on the cheek, nose, and forehead in different rotation angles, respectively. (**D**), (**E**), and (**F**) show that the UVR irradiance on the cheek, nose, and forehead changes with SEA in different rotation angles, respectively.

The diurnal variations of the ambient UVR irradiance as well as the cheek, nose, and forehead exposure UVR irradiances for different rotation angles are respectively shown in Figure 4A–C. The diurnal variations of the ambient UVR irradiance were bell-shaped curves with peaks at approximately noon (approximately 12:00 CST), and the ambient UVR irradiances were nearly the same for the two days. The maximum ambient UVR irradiance at noon was approximately 6000.36 μW cm^−2^. In contrast, the cheek, nose, and forehead exposure to UVR irradiance changed by rotation angles. For the 0–90° rotation angles, the diurnal variations of UVR at the three monitored facial locations were different from that of the horizontal ambient, which exhibited bimodal distributions with peak values in the morning (approximately 10:15 CST for cheek and approximately 9:50 CST for the nose and forehead) and in the afternoon (approximately 13:30 CST for the cheek and approximately 14:00 CST for the nose and forehead). The peak values of the UVR irradiance in the cheek, nose, and forehead in the morning were approximately 2010.39 μW cm^−2^, 2734.96 μW cm^−2^ and 3167.28 μW cm^−2^, respectively. In the afternoon, these values were approximately 1942.65 μW cm^−2^, 2682.08 μW cm^−2^ and 3089.34 μW cm^−2^. For the rotation angles of 90–180°, the diurnal variations of the UVR irradiance at the three monitored facial locations exhibited unimodal distributions with peaks around noon that were similar to those associated with the ambient UVR. However, the peak values of the UVR irradiance for the cheek, nose, and forehead on these rotation angles around noon were approximately 1445.66 μW cm^−2^, 1069.81 μW cm^−2^ and 1450.14 μW cm^−2^, which were much lower than that of other rotation angles in the morning and afternoon. 

Figure 4D–F show that for the rotation angles of 0–90°, the maximum UVR irradiances were obtained at a SEA of approximately 65° for the cheek. At a lower SEA (<65°), the cheek UVR and ambient UVR irradiance increased with an increase in SEA. At a SEA of 65–82°, the cheek UVR irradiance decreased as SEA increased. The maximum UVR irradiances were obtained at a SEA of approximately 60° for the nose and forehead. At higher rotation angles (96–180°), the UVR irradiances at three monitored facial locations all showed little variation and were relatively low. However, at rotation angles of 96–180°, the UVR irradiance at the three monitored facial locations increased with an increase in SEA, but the maximum UVR irradiance were much lower than that of 0–90° rotation angles. 

### 3.2. Exposure Ratio of the Three Monitored Facial Locations H_UVR_ to Ambient H_UVR_

From Table 1, we can see that the exposure ratios are different for different rotation angle ranges. For the same time period and facial locations, the exposure ratios in decreasing were 60°, 120°, and 180° rotation angle ranges facing the sun, 360° rotation angle range and 180° rotation angle range backing to the sun. Around noon (11:00–13:00 CST, SEA 73–82°), the exposure ratios were relatively stable, ranging from 0.24 to 0.31 (cheek), from 0.18 to 0.31 (nose), and from 0.20 to 0.40 (forehead) for all rotation angle ranges. However, besides noon, the exposure ratios were significantly different. The maximum exposure ratios for the 60°, 120°, and 180° rotation angle ranges facing the sun and 360° rotation angle ranges occurred within 7:00–8:00 CST (SEA 24–37°) for the cheek (corresponding to 0.47, 0.44, 0.41, and 0.36) and 16:00–17:00 CST (22–35° SEA) for the nose (corresponding to 0.88, 0.77, 0.65, and 0.49) and the forehead (corresponding to 0.97, 0.87, 0.75, and 0.55). The maximum exposure ratios for the 180° rotation angle range facing away from the sun occurred within 6:00–7:00 CST (SEA 11–24°) for the cheek (0.32) and 17:00–18:00 CST (SEA 22–10°) for the nose (0.39) and the forehead (0.43). The minimum exposure ratios for the 60°, 120°, and 180° rotation angle ranges facing the sun as well as the 360° rotation angle range and the 180 rotation angle range backing to the sun occurred at 10:00–11:00 CST (SEA 63–75°) or 13:00–14:00 CST (SEA 73–61°), corresponding to 0.34, 0.32, 0.29, 0.27, and 0.23 for the cheek; 0.43, 0.39, 0.35, 0.27, and 0.19 for the nose; as well as 0.52, 0.48, 0.43, 0.32, and 0.21 for the forehead, respectively.

### 3.3. UVR and UVBE_skin_ Changes with Rotation Angles

**Figure 5:**
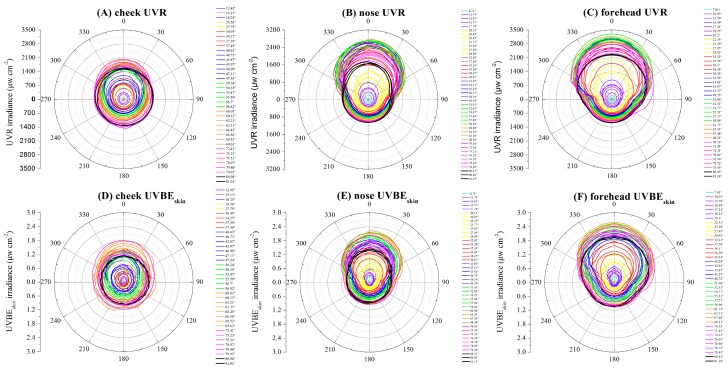
The UVR and UVBE_skin_ changes with rotation angles. Notes: (**A**), (**B**), and (**C**) shows the cheek, nose, and forehead UVR changes with rotation angles, respectively. (**D**), (**E**), and (**F**) show the cheek, nose, and forehead UVBE_skin_ changes with rotation angles, respectively.

The ambient UVR irradiance did not change with the rotation angle. However, the cheek UVR irradiance significantly changed with the rotation angle until the SEA was greater than 80° (see Figure 5A), while the nose and forehead UVR irradiance significantly changed with the rotation angle for all SEAs. As expected, the UVR irradiance at the three monitored facial locations decreased as the model rotated away from the direction of the sun. However, at a SEA of >80°, the cheek UVR irradiance did not change significantly with the rotation angle. Overall, the UVR irradiance at the three monitored facial locations exposure decreased as the model rotated away from the rotation angle of 0°. The maximum UVR irradiance occurred at the 0° position, while the minimum UVR irradiance at the 180° position. A significant decrease was found when the cheek and nose rotated from the 0° position to the 90° position in addition to when the forehead rotated from the 0° position to the 108° position, with a decline of approximately 52.70%, 52.10%, and 62.70% for the cheek, nose, and forehead, respectively. The UVR irradiance was relatively low and stable between the positions from 90° to 270° for the cheek and nose as well as from 108° to 252° for forehead. As shown in Figure 5A–C, the UVR exposure occurring at the 0° position increased as the SEAs increased to 70° for the cheek and 60° for the nose and forehead. The UVR irradiance at the three monitored facial locations exposure occurred at the 180° position were both increased as the SEA was increased. The cheek, nose, and forehead UVR irradiances at SEA = 53.6° (~1583.99 μW cm^−2^), 27° (~1663.7 μW cm^−2^), and 32° (~2241.6 μW cm^−2^) was as much as the exposure around noon at SEA = 82° (~1525.03 μW cm^−2^, 1649.7 μW cm^−2^, and 2241.6 μW cm^−2^, respectively). Figure 5D–F shows that the UVBE_skin_ trends for the three monitored facial locations are closer to UVR at the three monitored facial locations (Figure 5A–C). The peak UVBE_skin_ irradiance values for the cheek, nose, and forehead recorded at the 0° position are approximately 1.70 μW cm^−2^, 2.23 μW cm^−2^, and 2.60 μW cm^−2^, respectively. 

### 3.4. HBE_skin_ and RR Changes with Rotation Angles

**Figure 6:**
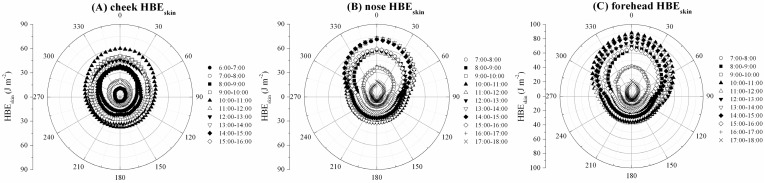
1 h cumulative HBE_skin_ changes with rotation angles: (**A**) cheek, (**B**) nose, and (**C**) forehead.

Figure 6 shows the 1 h cumulative cheek, nose, and forehead HBE_skin_ (in J m^−2^) with changes of the rotation angles from 6:00 to 14:00 CST for the cheek (Figure 6A), while the nose and forehead was measured at 7:00 to 18:00 CST (Figure 6B,C). The maximum and minimum 1 h cumulative cheek, nose, and forehead HBE_skin_ all occurred at the 0° position and 180° position, respectively. The peaks of 1 h cumulative cheek, nose, and forehead HBE_skin_ all occurred in the period of 10:00–11:00 CST in the morning, which were found to be approximately 50.14 J m^−2^, 72.92 J m^−2^, and 88.15 J m^−2^, respectively. The peaks of these values also occurred during the period of 13:00–14:00 CST in the afternoon, which were found to be approximately 42.64 J m^−2^, 72.98 J m^−2^, and 83.27 J m^−2^, respectively, with small differences found. However, for the same time period, the 1 h cumulative cheek, nose, and forehead HBE_skin_ at the 180° position was only approximately 31.25 J m^−2^, 31.94 J m^−2^, and 36.58 J m^−2^ in the morning, while it was found to be approximately 25.45 J m^−2^, 32.27 J m^−2^, and 32.59 J m^−2^ in the afternoon, respectively, with slight differences. 

**Figure 7:**
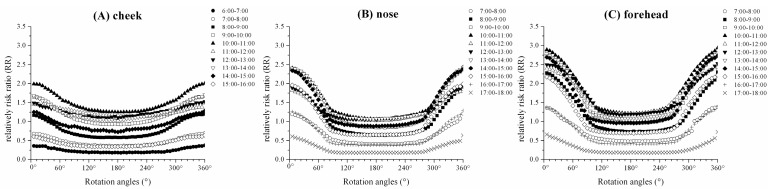
Relative risk ratios (RR) of 1 h cumulative HBE_skin_ relative to the ELs (30 J m^−2^): (**A**) cheek, (**B**) nose, and (**C**) forehead.

Figure 7A–C show the RRs of the 1 h cumulative cheek, nose, and forehead HBE_skin_ relative to the ELs (30 J m^−2^). For the cheek, nose, and forehead, the maximum RRs all occurred at the 0° position during all time periods. For the same rotation angles, the maximum RRs occurred at 10:00–11:00 CST, with values being approximately 2.01, 2.40, and 2.90 for the cheek, nose, and forehead, respectively. The RR was relatively low and stable between the rotation angles from 90 to 270°, with the maximum RRs being approximately 1.04 and 1.06 for cheek and nose at 10:00–11:00 CST. Between the rotation angles from 108 to 282°, and approximately 1.21 for the forehead at 10:00–11:00 CST. 

### 3.5. Percentage Difference of 1 h Cumulated HBE_skin_ to ELs

The increasing or decreasing percentages of the average cheek, nose, and forehead HBE_skin_ exposures over different rotation angle ranges compared to ELs (30 J m^−2^) are shown in Table 2. For the cheek and forehead, the maximal increasing percentage of the 60°, 120°, and 180° rotation angle ranges facing the sun as well as the 360° rotation angle ranges and the 180° rotation angle ranges backing to the sun all occurred in the period of 10:00–11:00 CST. These maximal percentages were 62.10%, 52.72%, 43.43%, 26.27%, and 8.59% for the cheek and 178.61%, 159.19%, 134.38%, 83.41%, and 30.79% for the forehead, respectively. For the nose, the maximal increasing percentage of the 60°, 120° facing the sun occurred in the period of 13:00–14:00 CST, and 130.61% and 109.68% respectively; 180° rotation angle ranges facing the sun as well as the 360° rotation angle ranges and the 180° rotation angle ranges backing to the sun occurred in the period of 10:00–11:00 CST, and 86.43%, 50.06%, and 12.09% respectively. The maximal decreasing percentage of the 60°, 120°, and 180° rotation angle ranges facing the sun as well as the 360° rotation angle ranges and the 180° rotation angle ranges backing to the sun occurred in the period of 6:00–7:00 CST for the cheek, which were found to be 70.63%, 73.13%, 75.72%, 79.63%, and 83.72%, respectively. For the nose and forehead, the maximal decreasing percentage of the 60°, 120°, and 180° rotation angle ranges facing the sun as well as the 360° rotation angle ranges and the 180° rotation angle ranges backing to the sun all occurred in the period of 17:00–18:00 CST, which were found to be 47.78% (nose) and 45.02% (forehead); 54.73% (nose) and 53.62% (forehead); 61.70% (nose) and 60.62% (forehead); 71.23% (nose) and 70.44% (forehead); and 81.32% (nose) and 80.77% (forehead), respectively.

## 4. Discussion

To study the health risk assessment of skin damage from UV irradiance for different rotation angles, a rotating model, and a spectroradiometer was used to monitor the face skin exposure and ambient UV irradiance at different rotation angles performed during two days with clear skies. The three selected facial locations on the face were the cheek, nose, and forehead. The API is a simple and generalized way to describe air quality, with a higher API corresponding to more serious air pollution. Some studies have shown that air pollution can reduce the surface levels of UV irradiance [39]. For both measuring days, (i) the mean API was approximately 62, (ii) the result of the diurnal variations of the ambient UVR irradiance were bell-shaped curves with peaks at approximately noon (approximately 12:00 CST, SEA 82°), and (iii) the ambient UVR irradiances were nearly the same for the two days. These facts proved that the distribution of UV irradiance was related to air quality on the monitoring days. 

This study shows that there were diurnal variations of UVR irradiances on the cheek, nose, and forehead, which exhibited bimodal distributions. For the rotation angles of 0–90°; the maximum UVR irradiances were obtained at SEA of approximately 65° for cheek as well as approximately 60° for nose and forehead. At a lower SEA (<65° for cheek, <60° for nose and forehead), the UVR irradiance increased with an increase in SEA. At a higher SEA (65–82° for cheek, 60–82° for nose and forehead), the UVR irradiance decreased as SEA increased. At higher rotation angles (96–180°), the UVR irradiances at the three facial monitoring locations all showed little variation and were relatively low. The critical values of SEA for the cheek, nose, and forehead all differed, which was mainly influenced by the surrounding anatomical structure. When the SEA was lower than a critical value, the facial locations received direct UV radiation as well as scattering and ground reflected UV, with the total UV exposure being increased by SEA. When the SEA was higher than a critical value of SEA, the facial locations only received scattering and ground reflected UV with a lack of direct UV, therefore resulting in the total UV irradiance being reduced. 

The biologically damaging effects of UV irradiance on human bodies are dependent on the biologically effective UV irradiance (UVBE). In this study, the peak skin damage from biologically effective UV irradiance (UVBE_skin_) values for cheek, nose, and forehead recorded at the rotation angle of 0° were approximately 1.70 μW cm^−2^, 2.23 μW cm^−2^ and 2.60 μW cm^−2^, respectively. The peaks of 1 h cumulative cheek, nose, and forehead skin damage from biologically effective UV radiant exposure (HBE_skin_) were all in the period of 10:00–11:00 CST in the morning, which were approximately 50.14 J m^−2^, 72.92 J m^−2^ and 88.15 J m^−2^, respectively. The peaks occurred during 13:00–14:00 CST in the afternoon, which had values of approximately 42.64 J m^−2^, 72.98 J m^−2^ and 83.27 J m^−2^, respectively. The ELs were used as guides in the control of exposure to UV sources and were developed by considering lightly pigmented populations (i.e., white Caucasian) with greatest sensitivity and genetic predisposition for skin cancer. We calculated the RRs of HBE_skin_ to ELs to assess the potential risk effects on skin caused by UV radiation. This study found that the maximum RRs for cheek, nose, and forehead at the 0° position were 2.01, 2.4, and 2.9 in the morning (10:00–11:00 CST), while these were 1.71, 2.43, and 2.78 in the afternoon (13:00–14:00 CST), respectively. However, the maximum average RR of 11:00–12:00 and 12:00–13:00 for the cheek, nose, and forehead at the 0° position were 1.51, 1.85, and 2.48, respectively. These results showed that the high-risk period for skin damage of the cheek, nose, and forehead was not at midday. This is in contrast to the WHO, which has shown that noon is a time of high risk of skin damage caused by UV radiation [40].

As we all know, during outdoor activities, the relative orientation of an individual’s facial locations to the sun can be random and is constantly changing. The results of the increasing or decreasing percentage of the average 1 h cumulative cheek, nose, and forehead HBE_skin_ from different rotation angle ranges to ELs can show average risks for different rotation angle ranges. In this study, the increasing percentage of cumulative UVBE_skin_ for 60°, 120°, 180°, and 360° rotation angle ranges facing the sun were maximal 62.10%, 52.72%, 43.43%, and 26.27% for the cheek; 130.61%, 109.68%, 86.43%, and 50.06% for the nose; and 178.61%, 159.19%, 134.38%, and 83.41% for the forehead, respectively. We found that in a rotation angle of 60° facing the sun, the cheek, nose, and forehead UV exposure declined significantly, especially for the period of 10:00–11:00 CST. Therefore, the rotation angle influenced the UVBE irradiance that the skin on the face is exposed to. The public should be prompted to better avoid the rotation angle of 60° facing the sun to reduce the biologically effective UV exposure and to reduce the risk of UV-related skin damage. 

Actually, the climatic conditions, the geographic variations, the weather conditions, API and surface albedo all influence the UV spectral irradiance and UV radiant exposure. This research was performed on sunny days in a low-latitude and low-altitude area in the northern hemisphere, while the measuring site was a concrete roof covered by asphalt, where the selected monitored facial locations of the model were fixed. Therefore, the results would be different for different conditions. Further, more research about skin UV exposure under different weather conditions, such as cloudy or air polluted skies, in other geographic areas of different surface albedos should be undertaken.

## 5. Conclusions

The risk of face skin exposure to UV irradiance was influenced by the rotation angle ranges relative to the sun.

## Figures and Tables

**Table 1:** The exposure ratios of the cheek, nose, and forehead after averaging H_UVR_ (in J m^−2^) for the different rotation angle ranges to the ambient H_UVR_ (in J m^−2^).

Facial Locations	SEA Range	Period	Ambient H_UVR_	360°	180° F	120° F	60° F	180° B
Cheek	11–24°	06:00–07:00	30,749.27	0.35	0.40	0.42	0.45	0.32
	24–37°	07:00–08:00	51,761.74	0.36	0.41	0.44	0.47	0.29
	37–50°	08:00–09:00	81,751.25	0.34	0.41	0.45	0.46	0.26
	50–63°	09:00–10:00	113,270.74	0.32	0.38	0.42	0.44	0.26
	63–75°	10:00–11:00	152,867.62	0.28	0.33	0.35	0.37	0.24
	75–82°	11:00–12:00	174,537.78	0.25	0.27	0.27	0.28	0.24
	82–73°	12:00–13:00	154,390.77	0.26	0.29	0.30	0.31	0.24
	73–61°	13:00–14:00	149,552.82	0.27	0.30	0.32	0.34	0.23
	61–48°	14:00–15:00	120,036.45	0.28	0.32	0.34	0.36	0.24
	48–35°	15:00–16:00	73,823.68	0.28	0.32	0.34	0.36	0.25
Nose	24–37°	07:00–08:00	68,949.99	0.45	0.58	0.66	0.73	0.31
	37–50°	08:00–09:00	111,943.17	0.40	0.54	0.63	0.71	0.25
	50–63°	09:00–10:00	151,350.58	0.34	0.45	0.52	0.58	0.22
	63–75°	10:00–11:00	188,380.90	0.27	0.35	0.39	0.43	0.20
	75–82°	11:00–12:00	212,581.23	0.22	0.25	0.27	0.30	0.18
	82–73°	12:00–13:00	211,343.41	0.22	0.26	0.28	0.31	0.18
	73–61°	13:00–14:00	187,553.73	0.27	0.35	0.40	0.44	0.19
	61–48°	14:00–15:00	152,975.64	0.34	0.46	0.54	0.60	0.21
	48–35°	15:00–16:00	109,329.52	0.43	0.60	0.71	0.80	0.25
	35–22°	16:00–17:00	66,603.76	0.49	0.65	0.77	0.88	0.31
	22–10°	17:00–18:00	29,628.03	0.49	0.59	0.66	0.72	0.39
Forehead	24–37°	07:00–08:00	68,949.99	0.50	0.64	0.71	0.78	0.37
	37–50°	08:00–09:00	111,943.17	0.46	0.63	0.73	0.81	0.29
	50–63°	09:00–10:00	151,350.58	0.40	0.55	0.63	0.69	0.25
	63–75°	10:00–11:00	188,380.90	0.33	0.43	0.48	0.52	0.23
	75–82°	11:00–12:00	212,581.23	0.28	0.34	0.37	0.39	0.21
	82–73°	12:00–13:00	211,343.41	0.28	0.35	0.38	0.40	0.21
	73–61°	13:00–14:00	187,553.73	0.32	0.43	0.48	0.53	0.21
	61–48°	14:00–15:00	152,975.64	0.39	0.55	0.63	0.69	0.24
	48–35°	15:00–16:00	109,329.52	0.49	0.68	0.80	0.89	0.28
	35–22°	16:00–17:00	66,603.76	0.55	0.75	0.87	0.97	0.35
	22–10°	17:00–18:00	29,628.03	0.54	0.64	0.70	0.78	0.43

“F” shows the rotation angle ranges facing the sun; “B” shows the rotation angle ranges backing to the sun.

**Table 2:** The increasing or decreasing percentages of average 1 h cumulative cheek, nose, and forehead HBE_skin_ from different rotation angle ranges compared to ELs (30 J m^−2^) (%).

Facial Locations	SEA Range	Period	360°	180° F	120° F	60° F	180° B
Cheek	11–24°	06:00–07:00	–79.63	–75.72	–73.13	–70.63	–83.72
	24–37°	07:00–08:00	–60.54	–52.88	–48.61	–44.84	–68.45
	37–50°	08:00–09:00	–33.95	–19.75	–11.74	–4.82	–48.64
	50–63°	09:00–10:00	–0.25	17.11	26.83	35.07	–18.15
	63–75°	10:00–11:00	26.27	43.43	52.72	62.10	8.59
	75–82°	11:00–12:00	4.59	10.97	14.13	17.49	–1.95
	82–73°	12:00–13:00	4.81	13.54	17.73	21.97	–4.07
	73–61°	13:00–14:00	5.15	18.46	26.12	34.66	–8.49
	61–48°	14:00–15:00	–22.10	–11.15	–5.16	0.94	–33.28
	48–35°	15:00–16:00	−63.40	−57.17	−53.93	−50.85	−69.81
Nose	24–37°	07:00–08:00	−36.14	−16.63	−4.39	6.97	−56.52
	37–50°	08:00–09:00	4.63	38.59	60.30	79.59	−30.88
	50–63°	09:00–10:00	34.56	76.29	102.26	124.22	−9.07
	63–75°	10:00–11:00	50.06	86.43	109.24	128.87	12.09
	75–82°	11:00–12:00	28.52	48.73	62.23	75.47	7.46
	82–73°	12:00–13:00	31.79	53.05	68.07	83.15	9.58
	73–61°	13:00–14:00	48.98	85.03	109.68	130.61	11.11
	61–48°	14:00–15:00	34.98	76.05	103.88	125.73	−8.28
	48–35°	15:00–16:00	6.31	43.20	68.05	87.82	−32.63
	35–22°	16:00–17:00	−37.48	−15.57	0.64	15.81	−60.70
	22–10°	17:00–18:00	−71.23	−61.70	−54.73	−47.78	−81.32
Forehead	24–37°	07:00–08:00	−28.42	−9.12	2.08	13.87	−48.53
	37–50°	08:00–09:00	22.24	63.66	87.67	109.13	−20.84
	50–63°	09:00–10:00	60.61	114.86	144.66	169.04	4.25
	63–75°	10:00–11:00	83.41	134.38	159.19	178.61	30.79
	75–82°	11:00–12:00	68.23	106.84	122.77	134.27	28.87
	82–73°	12:00–13:00	69.25	109.75	127.80	141.17	27.85
	73–61°	13:00–14:00	66.53	116.57	143.43	163.36	14.47
	61–48°	14:00–15:00	55.44	107.34	137.41	160.32	0.99
	48–35°	15:00–16:00	18.04	60.68	87.55	108.64	−26.88
	35–22°	16:00–17:00	−29.56	−3.35	13.34	28.03	−57.08
	22–10°	17:00–18:00	−70.44	−60.62	−53.62	−45.02	−80.77

“+” president the increasing percentage; “−” president the decreasing percentage; “F” and “B” president the same as Table 1.
